# Eye movement characteristics reflected fatigue development in both young and elderly individuals

**DOI:** 10.1038/s41598-018-31577-1

**Published:** 2018-09-03

**Authors:** Ramtin Zargari Marandi, Pascal Madeleine, Øyvind Omland, Nicolas Vuillerme, Afshin Samani

**Affiliations:** 1 Sport Sciences, Department of Health Science and Technology, Faculty of Medicine, Aalborg University, Aalborg, Denmark; 2 Univ. Grenoble Alpes, AGEIS, Grenoble, France; 3 Institut Universitaire de France, Paris, France; 4 Department of Occupational and Environmental Medicine, Danish Ramazzini Center, Aalborg University Hospital, Aalborg, Denmark

**Keywords:** Human behaviour, Predictive markers, Biomedical engineering

## Abstract

Fatigue can develop during prolonged computer work, particularly in elderly individuals. This study investigated eye movement characteristics in relation to fatigue development. Twenty young and 18 elderly healthy adults were recruited to perform a prolonged functional computer task while their eye movements were recorded. The task lasted 40 minutes involving 240 cycles divided into 12 segments. Each cycle consisted of a sequence involving memorization of a pattern, a washout period, and replication of the pattern using a computer mouse. The participants rated their perceived fatigue after each segment. The mean values of blink duration (BD) and frequency (BF), saccade duration (SCD) and peak velocity (SPV), pupil dilation range (PDR), and fixation duration (FD) along with the task performance based on clicking speed and accuracy, were computed for each task segment. An increased subjective evaluation of fatigue suggested the development of fatigue. BD, BF, and PDR increased whereas SPV and SCD decreased over time in the young and elderly groups. Longer FD, shorter SCD, and lower task performance were observed in the elderly compared with the young group. The present findings provide a viable approach to develop a computational model based on oculometrics to track fatigue development during computer work.

## Introduction

Fatigue is a multifaceted phenomenon^1^, referring to an inhibiting feeling during a sustained task coincident with the body requiring a resting period for recovery. Regulatory processes in the nervous system can emerge even without self-awareness of fatigue^2^. This unconscious regulation may involve significant reduction in performance during sustained mental or physical activities. Consequently, objective assessment of fatigue is an important issue, especially in human factors.

Today, a growing number of tasks involve human-computer interaction. In addition, the extension of working life is a major challenge in terms of elevated human error^3–5^, and health risks linked to stress and anxiety^6^. Fatigue development in sedentary computer work may be exacerbated by mental demands^7^. Research often shows that fatigue can be avoided by imposing appropriate work-pause/break regimes^3,8^. Thus, to implement an effective pausing regime at work, objective measures to track down the development of fatigue would be required. So far, several psychophysiological modalities, e.g. eye tracking^9^, neural signals and imaging^10,11^, and cardiac rhythms^10^, have been investigated during recent years to fulfil this aim.

Eye movement characteristics provide indirect access to cognitive processes, e.g. decision making^12^, attention^13^, and memory^14^. Particularly, the ocular events, e.g. saccades, blinks, fixations, and pupillary responses, involve different neural circuitries in connection with visuomotor information processing^15^. A number of studies^9,16,17^ have suggested that saccade peak velocity (SPV), saccade duration (SCD), fixation duration (FD), blink duration (BD), blink frequency (BF), and pupil dilation range (PDR) may be sensitive to mental load variation and fatigue (Table 1 presents definitions of the terms).

**Table 1:** The operational definitions^76^ used to compute the oculometrics.

Oculometrics	Abbreviation (unit)	Ocular event attribution	Operational definition (computation method)
Saccade Peak Velocity	SPV (°/s)	Saccade	Maximum of gaze velocity during a saccade
Saccade Duration	SCD^a^ (s)	Saccade	Number of samples of a saccade divided by the sampling frequency^b^
Fixation Duration	FD (s)	Fixation	Number of samples of a fixation divided by the sampling frequency
Blink Duration	BD (s)	Blink	Number of samples of a blink divided by the sampling frequency
Blink Frequency	BF (Hz)	Blink	Number of blink occurrences during a cycle^c^ divided by the duration of a cycle
Pupil Dilation Range	PDR (cm)	Pupillary response	The range of change in pupil dilation

^a^The saccade duration was abbreviated here as SCD, not to be confused with the standard deviation (*SD*). ^b^The sampling frequency in this study was 360 Hz. ^c^The cycle is described in the method section.

Saccade has been identified as a relevant ocular event to study fatigue. In a two-hour virtual simulation of a driving task^18^, both the duration and peak velocity of saccades decreased with time-on-task (TOT). Saccade peak velocity decreased with increasing TOT in a computer task involving a complex decision making process^19^, and in an air traffic control task^9^. Eye blink has been shown to be an informative ocular event enabling tracking of fatigue^20^. The duration and frequency of blinks were sensitive to change in mental load and fatigue levels^17,21^. Furthermore, pupillary responses were reported to be changed with TOT in a 2-back task^22^.

A key component in fatigue development is the duration of TOT, which can be specified based on the processing load of a mental task. For example, even in a seemingly short TOT (20 min) of performing a simple reaction time task (psychomotor vigilance test), significant increase in fatigue and decrease in performance were reported^23^. In addition, in a visual tracking task^24^ the performance of participants reduced significantly around 35 min after the onset of the task. Of note, this decrease in performance correlated with blinking and fixation-based oculometrics.

In the elderly population, fatigue and its cognitive components^25^ are less studied^26^. In addition, little is known about age-related differences in the oculometrics in response to increasing TOT. Longer BD and higher BF were observed due to physiological changes in eyelid structures and eye muscle fibre types^27^ in the process of aging. However, another study failed to observe such a difference in BF between young and elderly individuals^28^. Nonetheless, evidence suggest that aging *per se* leads to a decline in cognitive functioning^29^, most likely reflected in oculometrics.

We hypothesized that a prolonged mentally demanding task leading to fatigue development would be reflected in oculometrics. Particularly, we hypothesized that such alterations due to fatigue would be markedly prevalent in the elderly^29–31^. This study assessed the changes in blinks, saccades, fixations, and pupillary responses during TOT in a well-studied model of computer work involving various cognitive processes, e.g. memorization, verbal perception, recall, decision-making, attention, visual search, and eye-hand coordination.

## Results

### Oculometrics

**Figure 1:**
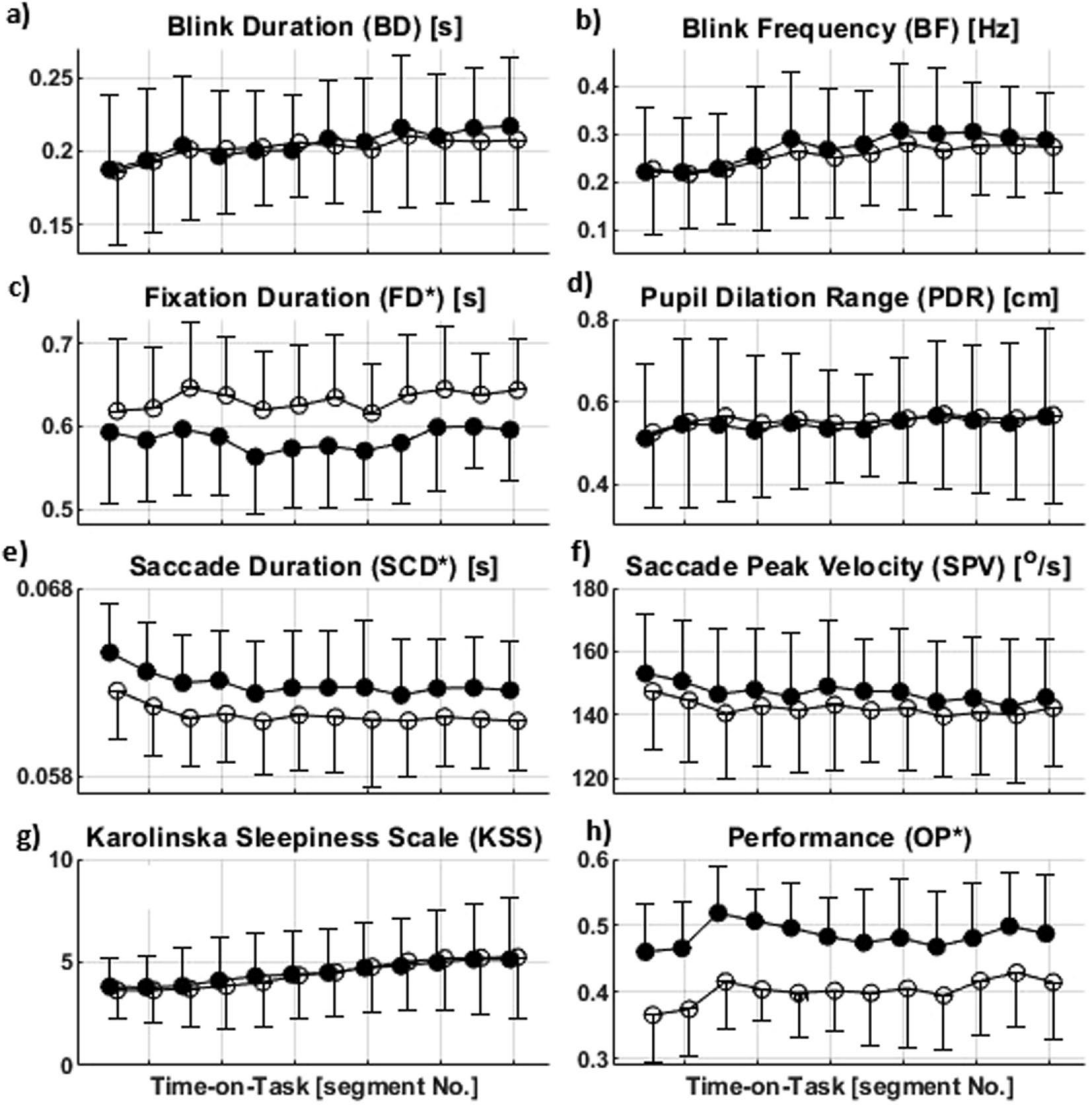
Illustration of the effect of time-on-task on the oculometrics (**a**–**f**) task performance (**h**) and fatigue ratings (**g**) (Mean and SD) in the young (black fill) and elderly group (white fill). The significant main effect of age is indicated by “*”, *p* < 0.05.

In both groups, BD increased with TOT, Fig. 1(a), $$F(6.2,198.6)=2.9,p=0.010,{\eta }_{p}^{2}=0.1$$. No significant difference was found between the groups in BD. In addition, there was no interaction of task segments and groups in BD. The BF also increased with TOT in the young and elderly groups, Fig. 1(b). $$F(3.7,118.9)=$$$$3.0,p=0.023,{\eta }_{{\rm{p}}}^{{\rm{2}}}=0.1$$. No significant difference was found between the young and elderly groups neither were there an interaction between the groups and the task segments. Post-hoc comparisons revealed that the changes in both BD and BF were significant between the first and the 11^th^ segments.

As illustrated in Fig. 1(d), PDR increased with TOT in the young and elderly groups, $$F(5.6,179.8)=$$$$3.0,p=0.009,{\eta }_{p}^{2}=0.1$$. No significant difference was found between age groups in PDR, neither an interaction between the groups and task segments. The significant differences were between the first segment and the segments of three, five, eight, and nine.

FD was affected by the TOT, $F(6.3,201.1)=2.5,p=0.019,{\eta }_{{\rm{p}}}^{2}=0.1$; however, no monotonic relationship between TOT and FD was found, Fig. 1(c). No interaction was found in FD between the groups and task segments. FD was higher in the elderly group compared with the young one, $F(1,32)=14.8,\,p=0.001,\,{\eta }_{p}^{2}=0.3$. Multiple comparisons revealed that there was a significant increase in FD from the first to the third segment among the elderly group.

SPV decreased with TOT in both groups, $$F(11,352)=4.6,p < 0.001,{\eta }_{p}^{2}=0.1$$, Fig. 1(f). No significant difference was found between groups, neither were there an interaction between the groups and task segments. There were significant changes from the first segment to the segments of 3–5, seven, eight, 10, and 11 according to the post-hoc comparisons. There was a main effect of TOT on SCD in both groups, $$F(4.8,153.1)=4.0,$$$$p=0.002,{\eta }_{{\rm{p}}}^{2}=0.1$$, Fig. 1(e). No interaction was found between the task segments and the groups in SCD. The young group had significantly higher SCD compared with the elderly group, $$F(1,32)=6.8,p=0.014,{\eta }_{{\rm{p}}}^{{\rm{2}}}=0.2$$. There were significant decreases from the first segment to the segments of three, five, and 9–11 in the young group.

The effect of TOT on the oculometrics remained significant even after introducing the averaged OP as a covariate to the statistical model. However, there were interactions between averaged OP and TOT in SCD, $$F(5.0,156.6)=$$ $$2.8,\,p=0.019,\,{\eta }_{p}^{2}=0.1$$ and between averaged OP and TOT in SPV, $$F(11,341)=70.7,$$$$p=0.022,\,{\eta }_{p}^{2}=0.1$$.

### Task performance

The overall performance (OP, see Methods) increased with TOT in the elderly group and had a fluctuating trend in the young one with an initial growth, $F(11,352)=4.3,p < 0.001,{\eta }_{{\rm{p}}}^{2}=0.1$, Fig. 1(h). There was no interaction between the groups and the task segments. OP was higher in the young group than the elderly group, $F(1,32)=3.0,p < 0.001,{\eta }_{{\rm{p}}}^{{\rm{2}}}=0.5$. OP was significantly higher in segments 10–12 compared with segment one in the elderly group. No significant differences in OP between segments were found in the young group.

### Subjective ratings

**Figure 2:**
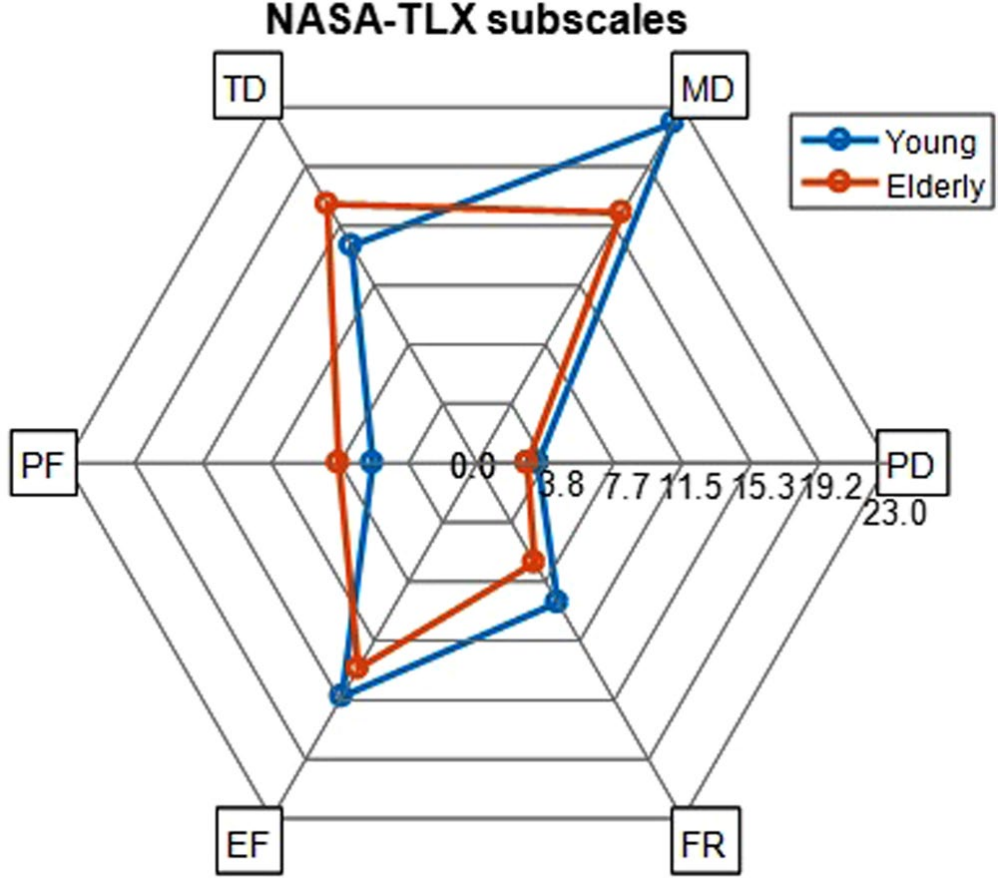
The radar chart of NASA-TLX scores for each workload subscale, i.e. MD: Mental Demand, PD: Physical Demand, FR: Frustration, EF: Effort, PF: Performance, and TD: Temporal Demand, in the young and elderly groups. After the fatigue block, the NASA-TLX was administered to specify the contribution of each workload subscale the participants perceived during the fatigue block. Both of the groups have found the task mentally and temporally demanding and not physically demanding.

The self-reported measures of fatigue, i.e. Karolinska Sleepiness Scale (KSS)^32^, increased with TOT in both the young and elderly groups as illustrated in Fig. 1(g), $F(1.9,65.4)=8.4,p=0.001,{\eta }_{p}^{2}=0.2$. No significant difference was found between the groups in KSS, neither were there an interaction between the task segments and groups. There were significant increases from segment one to the segments 7–12, from segment two to segment 7–12, from segment three to segment 8–12, from segment four to segment 7–12, from the segment five to segment 8–12 and, from segment six to segment 11. The effect of TOT on KSS remained significant after the adjustment for averaged OP. The results from National Aeronautics and Space Administration Task Load Index (NASA-TLX) questionnaire^33^ also confirmed that the participants have perceived the fatigue block (see Methods) mentally and temporally demanding, Fig. 2.

## Discussion

In the present work, the effects of a prolonged mentally demanding computer task on the oculometrics were investigated in both young and elderly individuals to address the role of aging in fatigue development during a functional task. As hypothesized, the blinking (BD and BF), fixation-based (FD), pupillary (PDR), and saccadic (SCD and SPV) oculometrics significantly changed with the progression of TOT in both groups, reflecting changes in the oculomotor control in response to sustained mental demands. Interestingly and contrary to our second hypothesis, the pattern of changes in oculometrics over time were quite similar in the young and elderly groups, suggesting that the underlying oculomotor control strategies used for the execution of the prolonged mentally demanding computer task were not remarkably affected by the aging process. This opens important perspectives for online feedback systems based on oculometrics.

The spontaneous blinks are known to be evoked to lubricate the surface of the cornea and conjunctiva. The blinking oculometrics, BD and BF, increased in both the young and the elderly group as TOT increased. These results are in agreement with previous findings on blinking in relation to fatigue^17,34,35^. The change in BF has been related to alterations in vigilance^36^ or arousal level^37^. The gradual loss of vigilance in sustained attentional tasks is usually accompanied by the co-occurrence of longer duration and higher rate of blinks^38^. The increase in BF can be described as the deactivation of blink inhibition as attentional resources decrease with fatigue development^39^. Prolonged BD may also be explained by changes in neural activation level of blink motoneurons in retractor bulbi and orbicularis oculi as fatigue progresses^39,40^. Thus, the co-occurrence of increased BF and BD most likely indicate fatigue development. The changes in the kinematics of blinks have shown that although amplitude and peak velocity of blinks change due to aging, blink rate may not change^41^. This may explain why no significant difference between the young and elderly groups was observed in BF.

The saccadic oculometrics, SPV and SCD, significantly decreased as TOT progressed in both groups. This is in accordance with previous findings^9,42,43^. The SCD and SPV followed a similar trend with TOT, which was expected due to the stereotyped linear relationship between the two parameters^18,44^. In this study, no significant difference was found between the young and elderly groups in SPV in agreement with some studies^45–47^. However, other studies^28,48^ have reported significant changes in saccadic eye movement characteristics due to aging. This can probably be explained by the fact that the computer task did not require saccade amplitude exceeding 20° ^48^. As some studies have suggested, elderly individuals have, compared with young adults, a higher tendency to move their head simultaneously with their eyes towards the visual stimulus during saccadic movements. Thus, to reach target points, elderly individuals are likely to make shorter saccades compared with young adults and compensate for the deficit by head movements^49^. The reduction of SCD and SPV across the task segments was not temporally aligned for individuals with high and low averaged OP, resulting in a significant interaction of averaged OP and TOT.

Fixations were also affected by the TOT in both the young and the elderly group. No monotonic pattern with increasing TOT was found, consistent with previous findings^50^. However, if the fixation duration is segmented into three classes of short (<150 ms), medium (<900 ms and >150 ms), and long (>900 ms), the duration of medium-length fixations increased with fatigue development in both the young and the elderly group. Some studies suggest that the medium-length fixations are associated with cognitive processing^39^. The higher FD in the elderly group compared with the young group could be explained by the decline in visual search efficacy due to aging^51^. In the elderly group, this may be an indicator of increased attentional efforts due to a decline in cognitive performance and working memory capacity, as suggested previously^52^.

In this study, PDR was found to increase as TOT increased for both groups. This is also in agreement with recent studies reporting an association between pupillary responses and fatigue development^22,53^. In sum, this supports the decline in perceptual performance with TOT, as pupil dilation increases in response to increased mental effort^54,55^.

Co-occurring changes in the oculometrics, i.e. a decrease in SPV together with an increase in PDR with TOT, have also been reported as a result of increase in mental load^16^. In addition, saccades and fixations having originated from distinct neural control circuits^56^, as well as the co-occurred changes in saccadic and fixation-based oculometrics found in the current study, may support that the fatigue development modulates activations in both of the distinct neural circuits. It thus suggests studying the similarity of sustained mental load and increasing mental demands of a task on oculomotor control strategies.

The effect of TOT on OP was observed in both groups. OP followed a fluctuating trend in the young and an increasing trend in the elderly group. It was higher in the young group than in the elderly group, most likely due to the loss of motor control efficacy with aging^47,57^, especially in computer tasks^58^. The performance during a mental task is not always expected to reduce with TOT^59^ as a compensatory mechanism may increase the allocated cognitive capacity to perform the task^60^. After adjusting the statistical model for the averaged OP, the main effect of TOT on the oculometrics and KSS remained significant, suggesting that the effect of TOT on the oculometrics and KSS was not mediated by the averaged OP.

Subjective ratings of fatigue, KSS, revealed that both groups perceived increasing levels of fatigue as TOT increased. Even though computer work may also involve physical demands, our results showed an association of fatigue development with mental demands and not with physical demands of computer work based on the subjective ratings of NASA-TLX^1^.

In this study, we imposed a specific level of mental demand in a cyclic computer task for 40 min. The repetitive pattern of computer work and an extended TOT were taken into account in the current study design since such specifications during long time may cause chronic or accumulated fatigue^61,62^. In a real-life scenario, a computer user may deal with different work patterns with varying levels of mental demands. Additionally, to allow for comparison of TOT across subjects, the randomly generated patterns were kept identical (See Methods). However, one may conceive that the non-monotonicity of temporal variation of oculometrics was due to the difference in the perceived difficulty of performing the task across the segments. To avoid such an effect, the geometrical features of the patterns were specifically set to keep a consistent level of complexity across the segments. The cognitive processes involved in computer work would also be different from task to task and may, in general, engage visual search, attention, and decision-making to a different extent. Thus, some general features, e.g. work pace, may provide a key factor to investigate the specificity and sensitivity of fatigue biomarkers such as the oculometrics in this study.

In conclusion, the current study investigated the effects of fatigue in a prolonged functional computer task. Despite the differences in SCD and FD between the young and elderly groups, all the oculometrics followed similar patterns of change over time. Compared with the fixation-based metrics, eye blinks, saccades, and pupillary responses revealed stronger relationships with TOT and may therefore be applicable to the tracking of fatigue development. The association of changing characteristics of eye movements with fatigue can potentially provide a quantitative approach to the development of an informed decision on a work-pause regime^63^ hampering fatigue.

## Methods

### Participants

Twenty participants, nine females and 11 males, aged 23 (*SD* 3) years old with the height of 1.74 (*SD* 0.08) m, and the body mass of 71 (*SD* 11) kg as a young group and 18 participants, 11 females and seven males, aged 58 (*SD* 7) with the height of 1.72 (*SD* 0.07) m, and the body mass of 80 (*SD* 12) kg as an elderly group were recruited. We had to set aside two young participants and one elderly participant from the study due to poor gaze recording quality. All participants had normal or corrected-to-normal vision (self-reported and examined by Snellen chart). They were familiar with computer work and used their right hand for computer mouse. The Edinburgh Handedness Inventory^64^ was administered to assess their handedness i.e. mean laterality index of 74 ± 40. Participants were asked for abstentions from alcohol for 24 h, and caffeine, smoking and drugs for 12 h prior to experimental sessions. The participants reported at least 6 h (mean 7.7 ± 1.1 h) of night sleep before the experimental session. In order to rule out the possibility of including participants suffering from chronic fatigue or eye strain symptoms, Fatigue Assessment Scale (FAS)^65^ and Visual Fatigue Scale (VFS)^66^ were administered respectively. The FAS includes 10 statements of fatigue and its psychological aspects. Each statement was answered on a 5-point scale (1, never to 5, always), thus the overall FAS score varies between 10 and 50. The VFS involves various symptoms of eyestrain which was rated on their occurrence frequency. The VFS scores ranged from zero to 60, respectively indicating none to all of the symptoms associated with the eyestrain. The resulting FAS and VFS ratings from the participants exhibited no symptom of fatigue-related disorders with 21 (*SD* 2) in FAS, and no eyestrain symptoms with seven (*SD* 5) in VFS. Written informed consent was obtained from each participant. The experiment was approved by The North Denmark Region Committee on Health Research Ethics, project number N-20160023 and conducted in accordance with the Declaration of Helsinki.

### Experimental task: A Functional Computer Task

**Figure 3:**
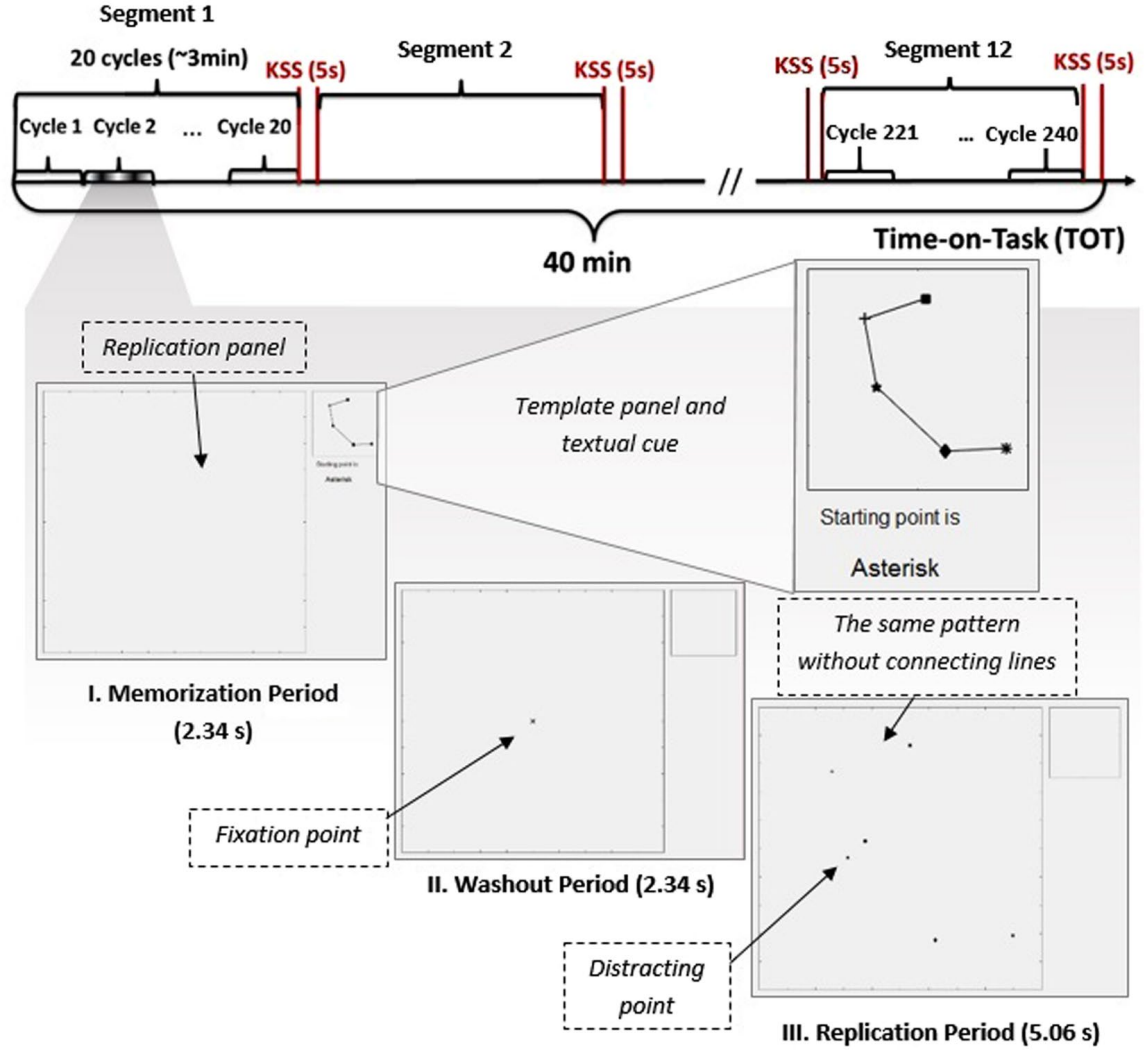
The timeline of fatigue block and the three sections constituting each cycle. The fatigue block included 240 cycles taking about 40 min. After each 20 cycles, a task segment, the participants rated their perceived fatigue on the Karolinska Sleepiness Scale (KSS) while the task execution was paused for five seconds. Each cycle began with the memorization period (I), then continued with the washout period (II), and ended with the replication period (III).

The task was designed and implemented in MATLAB R2015b (The Mathworks, Natick, MA) based on a standard model of computer work which has been used in previous studies^67,68^. The task was previously shown to be able to impose different levels of mental load^16^. The graphic user interface (GUI) of the computer task was displayed on a 19-in screen (1280 × 1024 pixels, refresh rate: 120 Hz) locating approx. 57 cm in front of the participant. The fixed size GUI (subtending approx. 27° horizontal and 22° vertically of visual angle) included a replication and a template panel, Fig. 3. The square shaped replication and template panel subtended approx. 20° and 5° of visual angle, respectively. The GUI was locked on the centre of the computer screen. The computer screen height was adjusted such that ear-eye line was approx. 15 degrees below the horizon when the participants sat upright and stared at the centre of the screen^69^.

The task consisted of cycles beginning with a memorizing a two dimensional pattern made by a sequence of five randomly-positioned points connected to each other by lines displayed on the template panel. Each point was depicted in a different shape (e.g., circle, square, and triangle) and a textual cue during memorization informed the subjects of the first point determining the order of connecting the points in the pattern. The cycle continued by a washout period during which the pattern disappeared. Meanwhile, the participant had to fixate on a cross, subtended 2° of visual angle, displayed on the centre of replication panel. The computer mouse cursor was invisible during the washout period to avoid any prepositioning. Subsequently, during the replication period, the points without connecting lines appeared on the replication panel, and the participant had to click on the points to replicate the pattern according to their order of succession. The first correct click was recognizable by doubling the point’s size; the rest correct clicks were recognizable by the appearance of a connecting line between the currently clicked point and the previous correct one. In addition to the points composing the pattern, a distracting point in a different shape from the constituent points appeared on the replication panel, which had not to be clicked by the participants. When the cycle’s period was elapsed, a new cycle with a different pattern appeared. The duration of memorization, washout and replication period were set to be 2.34, 2.34, and 5.06 s, respectively. This corresponded to a medium load setting^16^. In this setting, the angles in the patterns made by the five points were >60°, the distance between points was approximately in the range between 2° to 4° in the pattern presentation panel. In addition, no intersection in the generated patterns was allowed, and the centre of the patterns were aligned to the centre of the presentation panel to avoid perceptual bias across cycles. Even though randomly generated, the patterns were kept identical across participants, not to introduce differences on participant’s eye movements due to different patterns.

### Procedure

All experimental sessions were conducted between 10.00–12.00 a.m. and 1.00–3.00 p.m. to minimize the potential influence of circadian rhythm or diurnal variation. The participants were instructed about the task and practiced by performing five short training blocks (5-min) of the task with different levels of mental load as defined in^16^. After approx. 10-min rest, the participants were asked to perform the task containing 240 cycles for 40 min. After each 20 cycles, i.e. a task segment, the task execution paused for 5 s while the participants had to indicate their fatigue level on a 9-step Likert of Karolinska Sleepiness Scale (KSS)^32^ ranging from 1 to 9 respectively corresponding to “Very alert” and “Very sleepy, fighting sleep”. The participants also had to indicate their subjective perception of the mental load by answering the NASA-TLX questionnaire^33^ after the termination of the fatigue block. NASA-TLX provides an overall workload score based on a weighted average of ratings on six workload categories (mental demand, physical demand, temporal demand, performance, effort, and frustration). The participants were asked to perform the task as quick and accurate as they could. They were informed that their task performance will be measured and compared with the other participants (incentives on social comparison^70^). Furthermore, the participants were informed that the length of the experiment would be reduced and they would get a monetary reward if they persistently got a high task performance. Based on the preliminary results from our pilot studies, and to avoid any confounding factor such as pain and soreness from long time sitting, we chose the 40 min for the length of the TOT in current study.

### Data recording and analysis

A video-based monocular eye-tracker (Eye-Trac 7, Applied Science Laboratories, Bedford, MA, USA) was utilized to measure the eye movements and pupil diameter at a sampling frequency of 360 Hz. As reported by the manufacturer, spatial precision of the eye-tracker is lower than 0.5° of visual angle. The spatial accuracy is less than 2° in the periphery of the visual field. To have an empirical estimate of the accuracy throughout the recording, we calculated the spatial accuracy during the washout period of the task where the subjects were instructed to look at the centre of the replication panel. We found that the average accuracy was 0.7 ± 0.1° across participants and did not change across time (p > 0.05). The head movements were also tracked in 3-Dimensions using a motion tracker (Visualeyez II system set up with two VZ4000 trackers, Phoenix Technologies Inc., Canada). The data from eye tracker and motion tracker were coupled to compensate for head movements and to precisely estimate the point of gaze (built-in functionality of Eye-Trac 7). The calibration of the eye-tracker was performed before starting the tasks with 9-point calibration protocol. The calibration procedure was repeated if the nominal range of accuracy was not met. The experiments were conducted in an indoor and controlled environment to rule out potential confounding effects due to changes in ambient noise and illumination.

Saccades, blinks, and fixations using an adaptive data-driven algorithm^71^ were identified across the entire timeline of the fatigue block. The algorithm initiated by calculating the visual angle between consecutive coordinates of the point of gaze. Afterwards, the angular velocity and acceleration of the visual angle were derived using a second-order Savitzky–Golay filter^72^ with a length of 19 samples^71^. The saccades were extracted following the velocity and acceleration thresholding^71^ together with two additional criteria to detect onset and offset of saccades^73^. The two criteria remove the saccades where deviation from the main direction of saccade trajectories deviates from 60° or the change in intra-saccadic direction exceeds 40°.

The pupil diameter was pre-processed as a function of time by linear interpolation to estimate the missing samples during blinks. Then, a zero-phase low-pass Butterworth filter of order 3 was applied to remove the noise and artefacts usually occurring prior or after each blink^74^.

Blinks were detected when the pupil was closed for a duration of 50–700 ms. This range has been chosen to include the largest and shortest blinks as suggested by^73,75^. The range also was visually inspected on eye video image frames in 10 subjects. This range includes some blinks which might incorrectly be excluded if we limit the range to e.g. [100–400] ms. Noise samples were identified given these criteria: If the duration of closed eyelids (zero-valued samples of pupil dilation) was out of the blink duration range, or corneal reflection was not detected, or the gaze velocity or acceleration were higher than 500°/s and 50000°/s^2^, respectively. Excluding the instances of saccades, blinks, and noise samples, the remaining part of the gaze coordinates were considered as fixations if the duration of the detected fixation was >30 ms^76^. Successive fixations were merged into one fixation where <5 intra-fixation samples and <1° of visual dispersion between the preceding and succeeding fixations occurred^71,77^.

### Oculometrics

All the oculometrics in this study are stated in Table 1. PDR was computed to measure pupillary response, since it provides the absolute extent of change in pupil diameter. From saccadic and fixation-based events, the duration of saccades (SCD) and fixations (FD) as well as the peak velocity of saccades (SPV) were computed. Further, we segmented the fixations into three classes of short (<150 ms), medium (<900 ms and >150 ms), and long (>900 ms) as each class is suggested to reflect different neural activity^39^. In addition, BD and BF were computed to capture relevant information of fatigue progression from blinks. Based on the timestamps indicating the onset and offset of the task cycles, the detected ocular events were divided into their corresponding cycles. The cycles with less than five saccades and five fixations (minimum requirement to perform the task) were deemed invalid cycles and excluded from further processing (2 ± 3% of the cycles for each participant and task). The derived metrics were averaged across the valid cycles. To obtain a stable estimate of oculometrics and to span the saccades over a wide range from short saccades in the memorization period to long saccades in the replication period, the whole timeline of a cycle was considered for the calculation of oculometrics.

### Task Performance

The overall task performance (OP) was computed based on how accurate and fast the pattern replication was done. The ability to keep a set of actions in the face of distracting or competing stimuli^78,79^ was defined as selective attention (SelA). According to Table 2, a customized definition of SelA, and the mean reaction time (MRT) to complete each pattern was employed to compute the task performance. The MRT was divided by its minimum across all participants (i.e., 0.5) to assign the minimum normalized MRT to one. For each cycle, the task performance was computed as the ratio of the SelA to the normalized MRT. The SelA can become one in the highest attentive case when all the pattern points were clicked correctly and descended in low attentiveness. The OP theoretically is a positive value with zero for the lowest performance.

**Table 2:** The formula used to compute the task performance for each cycle.

Formula	Parameters
$$MRT=\{\begin{array}{ll}\frac{{\sum }_{i=1}^{CC}T{I}_{i}}{CC}, & {\rm{Completed}}\,{\rm{pattern}}\\ \frac{{\sum }_{i=1}^{CC}T{I}_{i}+RTRP}{CC+1}, & {\rm{Partially}}\,{\rm{completed}}\,{\rm{pattern}}\\ \mathrm{RP}, & {\rm{No}}\,{\rm{correct}}\,{\rm{clicks}}\end{array}$$	*TI*: Time interval between correct clicks (*TI* for the first correct click is computed from the replication period inset time) *CC*: Number of correct clicks *RP*: Replication period *IC*: number of incorrect clicks *PP*: Number of pattern points *DC*: number of clicks on the distracting point
$$SelA=\frac{CC}{IC+PP+DC}$$	

### Statistics

The oculometrics (SPV, SCD, FD, PDR), perceived fatigue ratings (KSS), and task performance (OP) were assessed for normality using Kolmogorov–Smirnov tests. We examined the effects of TOT (task segment 1 to 12) as within-subject factor, and the age (young and elderly groups) as between-subject factor, on the oculometrics, task performance, and fatigue ratings using a repeated measures analysis of variance. A Greenhouse-Geisser correction was applied if the assumption of sphericity was not met. The measure of effect size, partial eta-squared, ${{\rm{\eta }}}_{p}^{2}$, was also reported. A significant effect of TOT was further examined by post-hoc comparisons of task segments in pairs indicated by Bonferroni correction. Finally, to account for a probable mediating effect of performance on the TOT effect, we introduced averaged OP as the covariate to the statistical model. This was done following the recommendations for applying the analysis of covariance approach to a within-subjects design^80^. The statistical analysis was performed in SPSS 24.0. p < 0.05 was considered significant.

## References

[1] Enoka RM, Duchateau J (2016). Translating fatigue to human performance. Med. Sci. Sports Exerc..

[2] Ishii, A., Tanaka, M., Yoshikawa, T. & Watanabe, Y. Evidence for unconscious regulation of performance in fatigue. *Sci. Rep*. **7** (2017). 10.1038/s41598-017-16439-6 PMC5700951 29170440

[3] Weigl M, Antoniadis S, Chiapponi C, Bruns C, Sevdalis N (2015). The impact of intra-operative interruptions on surgeons’ perceived workload: an observational study in elective general and orthopedic surgery. Surg. Endosc..

[4] Techera U (2016). Measuring Occupational Fatigue: A Comprehensive Review and Comparison of Subjective and ObjectiveMethods. Proc. Constr. Res. Congr..

[5] Fisher GG, Chaffee DS, Tetrick LE, Davalos DB, Potter GG (2017). Cognitive functioning, aging, and work: A review and recommendations for research and practice. J. Occup. Health Psychol..

[6] Jepsen JR, Zhao Z, Van Leeuwen WMA (2015). Seafarer fatigue: a review of risk factors, consequences for seafarers’ health and safety and options for mitigation. Int. Marit. Health.

[7] Ahmed, S. Human fatigue in prolonged mentally demanding work-tasks: an observational study in the field. *156_Mississippi State Univ*. (2013).

[8] Luger, T., Maher, C. G., Rieger, M. A. & Steinhilber, B. Work-break schedules for preventing musculoskeletal disorders in workers. *Cochrane Database Syst. Rev*. (2017). 10.1002/14651858.CD012886.pub2 PMC6646952 31334564

[9] Di Stasi LL (2013). Microsaccade and drift dynamics reflect mental fatigue. Eur. J. Neurosci..

[10] Zhang, C. & Yu, X. Estimating mental fatigue based on electroencephalogram and heart rate variability. *Polish J. Med. Phys. Eng*. **16**, 67–84 (2010).

[11] Möckel, T., Beste, C. & Wascher, E. The Effects of Time on Task in Response Selection-An ERP Study of Mental Fatigue. *Sci. Rep*. **5** (2015). 10.1038/srep10113 PMC4460573 26054837

[12] Zargari Marandi, R. & Sabzpoushan, S. H. Qualitative modeling of the decision-making process using electrooculography. *Behav. Res. Methods* **47**, 1404–1412 (2015). 10.3758/s13428-014-0549-9 25515839

[13] Hopstaken JF, van der Linden D, Bakker AB, Kompier MA, Leung YK (2016). Shifts in attention during mental fatigue: Evidence from subjective, behavioral, physiological, and eye-tracking data. Journal of Experimental Psychology: Human Perception and Performance.

[14] Marandi, R. Z. & Sabzpoushan, S. H. Using eye movement analysis to study auditory effects on visual memory recall. *Basic Clin. Neurosci*. **5**, 55–65 (2014). PMC4202595 25436085

[15] Keller GB, Bonhoeffer T, Hübener M (2012). Sensorimotor Mismatch Signals in Primary Visual Cortex of the Behaving Mouse. Neuron.

[16] Marandi RZ, Madeleine P, Omland Ø, Vuillerme N, Samani A (2018). Reliability of Oculometrics During a Mentally Demanding Task in Young and Old Adults. IEEE Access.

[17] Stern JA, Boyer D, Schroeder D (1994). Blink rate: a possible measure of fatigue. Hum. Factors.

[18] Di Stasi LL (2012). Towards a driver fatigue test based on the saccadic main sequence: A partial validation by subjective report data. Transp. Res. Part C Emerg. Technol..

[19] Di Stasi LL, Antolí A, Cañas JJ (2013). Evaluating mental workload while interacting with computer-generated artificial environments. Entertain. Comput..

[20] Martins, R. & Carvalho, J. In *Occupational Safety and Hygiene III* 231–235 (2015).

[21] Borghini G, Astolfi L, Vecchiato G, Mattia D, Babiloni F (2014). Measuring neurophysiological signals in aircraft pilots and car drivers for the assessment of mental workload, fatigue and drowsiness. Neurosci. Biobehav. Rev..

[22] Hopstaken JF, van der Linden D, Bakker AB (2015). & Kompier, M. A. J. The window of my eyes: Task disengagement and mental fatigue covary with pupil dynamics. Biol. Psychol..

[23] Lim J (2010). Imaging brain fatigue from sustained mental workload: An ASL perfusion study of the time-on-task effect. Neuroimage.

[24] Van Orden KF, Jung T-P, Makeig S (1998). Eye Activity Correlates of Fatigue during a Visual Tracking Task. Proc. Hum. Factors Ergon. Soc. Annu. Meet..

[25] Smith, B. T. The Effect of Situational and Individual Factors on Young and Older Adults’ Cognitive Fatigue. (Doctoral Dissertation). (North Carolina State University, 2016).

[26] Liao S, Ferrell Ba (2000). Fatigue in an older population. J. Am. Geriatr. Soc..

[27] Cavallotti, C. & Cerulli, L. *Age-related changes of the human eye*. Springer Science & Business Media, (2008).

[28] Dowiasch S, Marx S, Einhäuser W, Bremmer F (2015). Effects of aging on eye movements in the real world. Front. Hum. Neurosci..

[29] Salthouse TA (2009). When does age-related cognitive decline begin?. Neurobiol. Aging.

[30] Andersen GJ (2012). Aging and vision: Changes in function and performance from optics to perception. Wiley Interdiscip. Rev. Cogn. Sci..

[31] Czaja SJ, Sharit J (1993). Age Differences in the Performance of Computer-Based Work. Psychol. Aging.

[32] Åkerstedt T, Gillberg M (1990). Subjective and Objective Sleepiness in the Active Individual. Int. J. Neurosci..

[33] Hart, S, G. NASA-task load index (NASA-TLX); 20 years later. *Hum. Factors Ergon. Soc. Annu. Meting* 904–908 (2006).

[34] Fazio R, Coenen C, Denney RL (2012). The original instructions for the Edinburgh Handedness Inventory are misunderstood by a majority of participants. Laterality Asymmetries Body, Brain Cogn..

[35] Maffei, A. & Angrilli, A. Spontaneous eye blink rate: An index of dopaminergic component of sustained attention and fatigue. *Int. J. Psychophysiol*. **123**, 58–63 (2018). 10.1016/j.ijpsycho.2017.11.009 29133149

[36] Bodala IP, Li J, Thakor NV, Al-Nashash H (2016). EEG and Eye Tracking Demonstrate Vigilance Enhancement with ChallengeIntegration. Front. Hum. Neurosci..

[37] Di Stasi LL, Catena A, Cañas JJ, Macknik SL, Martinez-Conde S (2013). Saccadic velocity as an arousal index in naturalistic tasks. Neuroscience and Biobehavioral Reviews.

[38] McIntire LK, McKinley RA, Goodyear C, McIntire JP (2014). Detection of vigilance performance using eye blinks. Appl. Ergon..

[39] Schleicher R, Galley N, Briest S, Galley L (2008). Blinks and saccades as indicators of fatigue in sleepiness warnings: looking tired?. Ergonomics.

[40] Meinold, P. E. *Psychologie des Lidschlags-eine literatur-und methodenkritische Studie*. (Universität zu Köln, 2005).

[41] Sun WS (1997). Age-related changes in human blinks: Passive and active changes in eyelid kinematics. Investig. Ophthalmol. Vis. Sci..

[42] Di Stasi LL (2014). Saccadic Eye Movement Metrics Reflect Surgical Residents’ Fatigue. Ann. Surg..

[43] Di Stasi LL (2016). Effects of long and short simulated flights on the saccadic eye movement velocity of aviators. Physiol. Behav..

[44] Bahill AT, Clark MR, Stark L (1975). The Main Sequence, A Tool for Studying Human Eye Movements. Math. Biosci..

[45] Hotson JR, Steinke GW (1988). Vertical and Horizontal Saccades in Aging and Dementia: Failure to Inhibit Anticipatory Saccades. Neuro-Ophthalmology.

[46] Abel LA, Troost BT, Dell’Osso LF (1983). The effects of age on normal saccadic characteristics and their variability. Vision Res..

[47] Munoz DP, Broughton JR, Goldring JE, Armstrong IT (1998). Age-related performance of human subjects on saccadic eye movement tasks. Exp. Brain Res..

[48] Moschner C, Baloh RW (1994). Age-related changes in visual tracking. J. Gerontol..

[49] Proudlock FA, Shekhar H, Gottlob I (2004). Age-related changes in head and eye coordination. Neurobiol. Aging.

[50] Wohleber, R. W. *The impact of automation reliability and fatigue on reliance*. (Doctoral dissertation). (University of Central Florida., 2016).

[51] Ho G, Scialfa CT, Caird JK, Graw T (2001). Visual search for traffic signs: the effects of clutter, luminance, and aging. Hum. Factors.

[52] Meghanathan, R. N., van Leeuwen, C. & Nikolaev, A. R. Fixation duration surpasses pupil size as a measure of memory load in free viewing. *Front. Hum. Neurosci*. **8** (2015). 10.3389/fnhum.2014.01063 PMC4301010 25653606

[53] Hopstaken JF, van der Linden D, Bakker AB, Kompier MAJ (2015). A multifaceted investigation of the link between mental fatigue and task disengagement. Psychophysiology.

[54] Pomplun, M. & Sunkara, S. Pupil Dilation as an Indicator of Cognitive Workload in Human-Computer Interaction. In *Human-centered computing: Cognitive, social and ergonomic aspects. Vol. 3 of the Proceedings of the 10th International Conference on Human-Computer Interaction* 542–546 (2003).

[55] Granholm E, Asarnow RF, Sarkin AJ, Dykes KL (1996). Pupillary responses index cognitive resource limitations. Psychophysiology.

[56] Shadmehr Reza (2017). Distinct neural circuits for control of movement vs. holding still. Journal of Neurophysiology.

[57] Seidler RD (2010). Motor control and aging: Links to age-related brain structural, functional, and biochemical effects. Neuroscience and Biobehavioral Reviews.

[58] Smith MW, Sharit J, Czaja SJ (1999). Aging, motor control, and the performance of computer mouse tasks. Hum. Factors.

[59] Ackerman, P. L. *Cognitive fatigue: Multidisciplinary perspectives on current research and future applications*. (American Psychological Association, 2011).

[60] Wang C, Trongnetrpunya A, Samuel IBH, Ding M, Kluger BM (2016). Compensatory Neural Activity in Response to Cognitive Fatigue. J. Neurosci..

[61] Tanaka M, Watanabe Y (2010). A new hypothesis of chronic fatigue syndrome: Co-conditioning theory. Med. Hypotheses.

[62] Ishii A, Tanaka M, Watanabe Y (2014). Neural mechanisms of mental fatigue. Reviews in the Neurosciences.

[63] Samani A, Holtermann A, Søgaard K (2009). & Madeleine, P. Effects of eccentric exercise on trapezius electromyography during computer work with active and passive pauses. Clin. Biomech..

[64] Oldfield RC (1971). The assessment and analysis of handedness: The Edinburgh inventory. Neuropsychologia.

[65] Michielsen HJ, De Vries J, Van Heck GL (2003). Psychometric qualities of a brief self-rated fatigue measure: The Fatigue Assessment Scale. J. Psychosom. Res..

[66] Heuer H, Hollendiek G, Kröger H, Römer T (1989). Die Ruhelage der Augen und ihr Einfluss auf Beobachtungsabstand und visuelle Ermuedung bei Bildschirmarbeit. Z Exp Angew Psychol.

[67] Birch L, Arendt-Nielsen L, Graven-Nielsen T, Christensen H (2001). An investigation of how acute muscle pain modulates performance during computer work with digitizer and puck. Appl. Ergon..

[68] Samani, A., Holtermann, A., Søgaard, K. & Madeleine, P. Active pauses induce more variable electromyographic pattern of the trapezius muscle activity during computer work. *J. Electromyogr. Kinesiol*. **19**, 430–437 (2009). 10.1016/j.jelekin.2008.11.011 19135388

[69] Kroemer, K. H. E., Kroemer, H. B. & Kroemer-Elbert, K. E. *Ergonomics: how to design for ease and efficiency*. (Pearson College Division, 2001).

[70] Boksem MAS, Meijman TF, Lorist MM (2006). Mental fatigue, motivation and action monitoring. Biol. Psychol..

[71] Nyström M, Holmqvist K (2010). An adaptive algorithm for fixation, saccade, and glissade detection in eyetracking data. Behav. Res. Methods.

[72] Savitzky A, Golay MJE (1964). Smoothing and Differentiation of Data by Simplified Least Squares Procedures. Anal. Chem..

[73] Larsson L, Nystrom M, Stridh M (2013). Detection of saccades and postsaccadic oscillations in the presence of smooth pursuit. IEEE Trans. Biomed. Eng..

[74] Pashler H (2014). Using Task-Induced Pupil Diameter and Blink Rate to Infer Cognitive Load *A Handb*. Human–Computer Interaction.

[75] Caffier PP, Erdmann U, Ullsperger P (2003). Experimental evaluation of eye-blink parameters as a drowsiness measure. Eur. J. Appl. Physiol..

[76] Holmqvist, K. *et al*. *Eye tracking: A comprehensive guide to methods and measures*. (OUP Oxford, 2011).

[77] Hessels, R. S., Niehorster, D. C., Kemner, C. & Hooge, I. T. C. Noise-robust fixation detection in eye movement data: Identification by two-means clustering (I2MC). *Behav. Res. Methods* 1–22 (2016). 10.3758/s13428-016-0822-1 PMC5628191 27800582

[78] Green C, Bavelier D (2003). Action video game modifies visual selective attention. Nature.

[79] Moran J, Desimone R (1985). Selective attention gates visual processing in the extrastriate cortex. Science.

[80] Hoyle, R. H. & Robinson, J. C. *Mediated and moderated effects in social psychological research*. (Handbook of methods in social psychology, 2004).

