# Author Correction: Eye movement characteristics reflected fatigue development in both young and elderly individuals

**DOI:** 10.1038/s41598-020-60914-6

**Published:** 2020-02-27

**Authors:** Ramtin Zargari Marandi, Pascal Madeleine, Øyvind Omland, Nicolas Vuillerme, Afshin Samani

**Affiliations:** 1 Sport Sciences, Department of Health Science and Technology, Faculty of Medicine, Aalborg University, Aalborg, Denmark; 2 Univ. Grenoble Alpes, AGEIS, Grenoble, France; 3 Institut Universitaire de France, Paris, France; 4 Department of Occupational and Environmental Medicine, Danish Ramazzini Center, Aalborg University Hospital, Aalborg, Denmark

Correction to: *Scientific Reports* 10.1038/s41598-018-31577-1, published online 03 September 2018

The article contains errors in Table 1, Table 2, Figure 1 and in the Reference list.

In Table 1 and Figure 1, the unit for PDR incorrectly reads “cm” and should read “mm”. The correct Table 1 and Figure 1 appear below.

**Table 1:** The operational definitions used to compute the oculometrics. (1) The saccade duration was abbreviated here as SCD, not to be confused with the standard deviation (SD). (2) The sampling frequency in this study was 360 Hz. (3) The cycle is described in the method section.

Oculometrics	Abbreviation (unit)	Ocular event attribution	Operational definition (computation method)
Saccade Peak Velocity	SPV (°/s)	Saccade	The maximum of the gaze velocity during the course of a saccade
Saccade Duration	SCD^1^ (s)	Saccade	Number of samples of a saccade divided by the sampling frequency^2^
Fixation Duration	FD (s)	Fixation	Number of samples of a fixation divided by the sampling frequency
Blink Duration	BD (s)	Blink	Number of samples of a blink divided by the sampling frequency
Blink Frequency	BF (Hz)	Blink	Number of blink occurrences during a cycle^3^ divided by the duration of a cycle
Pupil Dilation Range	PDR (mm)	Pupillary response	The range of pupil dilation

**Figure 1:**
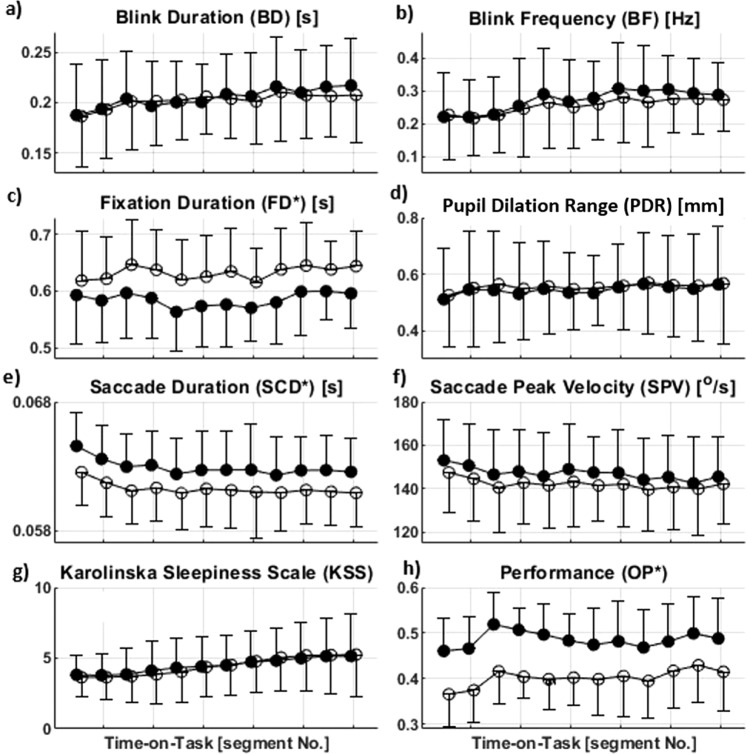
.

In Table 2, in the parameters column,

“*TI*: Time interval between correct clicks (*TI* for the first correct click is computed from the replication period inset time) *CC*: Number of correct clicks *RP*: Replication period *IC*: number of incorrect clicks *PP*: Number of pattern points *DC*: number of clicks on the distracting point”

should read:

“*TI*: Time interval between correct clicks (*TI* for the first correct click is computed from the replication period onset time) *CC*: Number of correct clicks *RP*: Replication period *IC*: number of incorrect clicks *PP*: Number of pattern points *DC*: number of clicks on the distracting point *RTRP*: Remaining Time of the RP”

The correct Table 2 appears below.

**Table 2:** The formula used to compute the task performance for each cycle.

Formula	Parameters
$$MRT=\{\begin{array}{ll}\frac{{\sum }_{i=1}^{CC}T{I}_{i}}{CC}, & {\rm{Completed}}\,{\rm{pattern}}\\ \frac{{\sum }_{i=1}^{CC}T{I}_{i}+RTRP}{CC+1}, & {\rm{Partially}}\,{\rm{completed}}\,{\rm{pattern}}\\ {\rm{RP}}, & {\rm{No}}\,{\rm{correct}}\,{\rm{clicks}}\end{array}$$	*TI*: Time interval between correct clicks (*TI* for the first correct click is computed from the replication period onset time) *CC*: Number of correct clicks *RP*: Replication period *IC*: Number of incorrect clicks *PP*: Number of pattern points *DC*: Number of clicks on the distracting point *RTRP*: Remaining Time of the RP
$$SelA=\frac{CC}{IC+PP+DC}$$	

Finally, in the Reference list, the title is missing from Reference 20. The correct Reference 20 appears below as Reference 1.
